# A Methodological Framework for Assessing the Influence of Process Parameters on Strand Stability and Functional Performance in Fused Filament Fabrication

**DOI:** 10.3390/ma16247530

**Published:** 2023-12-06

**Authors:** Eleni Gkartzou, Artemis Kontiza, Konstantinos Zafeiris, Elena Mantzavinou, Costas A. Charitidis

**Affiliations:** Research Lab of Advanced, Composite, Nano-Materials and Nanotechnology (R-NanoLab), School of Chemical Engineering, National Technical University of Athens, 9 Heroon Polytechniou, GR 15780 Athens, Greece; egartzou@chemeng.ntua.gr (E.G.); akontiza@chemeng.ntua.gr (A.K.); kzafeiris@chemeng.ntua.gr (K.Z.);

**Keywords:** additive manufacturing, conductive filaments, fused filament fabrication, electrical resistivity, micro-computed tomography

## Abstract

With an ever-increasing material and design space available for Fused Filament Fabrication (FFF) technology, fabrication of complex three-dimensional structures with functional performance offers unique opportunities for product customization and performance-driven design. However, ensuring the quality and functionality of FFF-printed parts remains a significant challenge, as material-, process-, and system-level factors introduce variability and potentially hinder the translation of bulk material properties in the respective FFF counterparts. To this end, the present study presents a methodological framework for assessing the influence of process parameters on FFF strand stability and functional performance through a systematic analysis of FFF structural elements (1D stacks of FFF strands and 3D blocks), in terms of dimensional deviation from nominal geometry and resistivity, corresponding to the printability and functionality attributes, respectively. The influence of printing parameters on strand stability was investigated in terms of dimensional accuracy and surface morphology, employing optical microscopy and micro-computed tomography (mCT) for dimensional deviation analysis. In parallel, electrical resistance measurements were carried out to assess the effect of different process parameter combinations and toolpath patterns on functional performance. In low-level structural elements, strand height (H) was found to induce the greatest influence on FFF strand dimensional accuracy and resistivity, with higher H values leading to a reduction in resistivity of up to 38% in comparison with filament feedstock; however, this occurred at the cost of increased dimensional deviation. At higher structural levels, the overall effect of process parameters was found to be less pronounced, indicating that the translation of 1D strand properties to 3D blocks is subject to a trade-off due to competing mechanisms that facilitate/hinder current flow. Overall, the proposed framework enables the quantification of the influence of process parameters on the selected response variables, contributing to the development of standard operating procedures and recommendations for selecting optimal process parameters to achieve the desired process stability and functional performance in FFF.

## 1. Introduction

Additive manufacturing (AM) is widely acknowledged for its potential to deploy new approaches, structures, and functions, offering an expanded material and design space for the fabrication of complex three-dimensional structures as well as unique opportunities for product customization and performance-driven design. AM has gradually been adopted for applications in automotive, aerospace, biomedical, energy, and customized consumer products industries [1]. Research efforts have focused on developing new high-performance materials and improving the efficiency and speed of the process, aiming to extend its impact on the fabrication of end products and better serve a wider range of industries that currently hesitate to adopt AM technologies [2,3,4]. However, ensuring the quality and functional performance of AM parts remains a significant challenge, as material-, process-, and system-level factors introduce variability and potentially hinder the translation of bulk material functional properties in the respective AM counterparts, thus rendering decision making for AM process and part design a non-trivial challenge [5]. In parallel, various manufacturing industries and application domains have developed extensive databases of material properties, standards, and specifications for conventional manufacturing technologies which have undergone years of development. This form of consolidated knowledge is missing from the AM industry, as there is still a gap to close in terms of development, standardization, and qualification of materials and processes [6].

Fused filament fabrication (FFF), also known under the general term of material extrusion AM, is one of the most popular AM processes that involves the selective deposition of thermoplastic feedstock in filament form. FFF is particularly versatile regarding material compatibility, spanning many polymer types, colors, and functionalities [7]. Passing through a liquefier, the filament feedstock is heated to a viscous melt and pushed through a print nozzle by the incoming, still-solid filament fraction. Extrudates in the form of compressed thermoplastic strands are then deposited onto the build platform according to predefined toolpaths and bond with adjacent strands to form a thin layer that rapidly solidifies [8]. The formation of bonds among individual strands and consecutive layers arises from complex heat and mass transfer phenomena coupled with thermal and mechanical stress accumulation and phase changes, thus resulting in highly anisotropic properties [9]. Although FFF is evolving into a manufacturing tool with significant technological and material advancements, it remains a challenge to transfer FFF-printed parts into functional objects for practical applications, with limited repeatability and precision frequently reported [10,11].

Many research works were conducted in the last decade to investigate the effect of process parameters on the quality of FFF parts in terms of surface roughness, dimensional accuracy, and mechanical properties, resulting in several property enhancement approaches through preprocessing methods (optimization of toolpath planning and process parameters) as well as postprocessing techniques (physical/chemical smoothening, thermal annealing) [10,12,13,14]. AM quality monitoring techniques have also been used to identify irregularities and forecast quality of the final part [15], although in the vast majority of desktop FFF systems, the open loop control of the process requires an experimental comparison of actual/nominal geometrical features. In process optimization, extrusion temperature, infill percentage and type, infill and specimen orientation, printing speed, extrusion width, and layer height have been studied extensively. However, multiple sources of uncertainty in each stage of process design and manufacturing have been identified, making it challenging to establish common ground for a comparative assessment between research outcomes. For instance, alterations in filament feedstock and 3D-printed part performance have been observed in virgin polymer materials, with modifications in the pigmentation, filament manufacturer, or FFF system employed [16,17]. In addition, the wide range of FFF systems and settings create variability in printed mechanical properties, with properties variations in terms of ultimate tensile strength (UTS) and yield strength reported, among others [18]. Furthermore, automatic toolpath generation by slicing software introduces sub-perimeter voids and principal stress directions with different spatial configurations, depending on the toolpath planning algorithms employed [9,19]. In this context, the challenges in process optimization and repeatability, within the complex FFF parameter space, have impacted FFF scalability in functional part production [7].

More recently, research efforts have focused on the development of more holistic, methodological frameworks, from printer calibration to slicer settings adjustment and postprocessing, aiming to reduce effects from uncontrolled factors [7,10,14,19,20]. Moreover, data-driven workflows that encompass knowledge management and concurrent optimization of design and process parameters have been proposed, also aiming to account for multiple sources of uncertainty [5,21,22]. In parallel, in order to simplify the complexity and clearly exhibit cause–effect relationships, a shift towards more fundamental studies has been proposed, for the investigation of FFF structural elements (single strands, 2D monolayers, and 3D blocks), to provide further insight on the translation of bulk material properties in their respective FFF counterparts. A key enabler in this pursuit is the development of complementary toolpath and process design tools that allow one to precisely define toolpath settings and couple the designed geometry with the manufacturing procedure [23]. To this end, recent experimental and numerical simulation studies have been devoted to evaluating stability and deformations of deposited strands and layers, as well as inter/intra-layer bond formation, in correlation with material- and process-specific aspects [11,13,24,25,26,27,28,29,30].

In more detail, for constant nozzle temperature, the melting duration of feedstock in the printhead is determined by printing speed [9]. Higher printing speeds translate into smaller windows for heat transfer, while the amount of time the extrudates retain temperatures above their glass transition has been found to be inversely proportional to printing speed. Successful bonding between adjacent strands is similar to the non-isothermal welding of polymer films, involving surface contact, neck growth, and molecular diffusion and entanglement across the inter-strand interface, with the equivalent time for the bonding being on the order of a few seconds [9,25,31]. Therefore, the longer contact time from slower printing speeds can facilitate interface welding and eliminate surface deformations associated with increased shear rates and inlet velocities [32]. Layer height and extrusion width are two key parameters to consider when optimizing FFF prints. Layer height is a determinant for slicing and build time, while extrusion width affects strand contact areas. Several competing mechanisms can be associated with the effect of the two factors on inter/intra-layer adhesion, as reduction in the layer height and/or extrusion width decreases the cross-sectional area of the deposited strand, thus leading to faster cooling. Moreover, as the values of the two factors decrease, the number of the required layers or XY passes increases, thus affecting the periodical temperature fluctuation with the deposition of a new layer which promotes chain diffusion; however, this occurs at the cost of the accumulation of residual stresses [26,28]. It was also reported that at smaller layer heights, bond quality is dominated by the cooling rate-driven mechanism, while at larger layer heights, contact pressure controls the bond interface [26]. In addition, the influence of extrusion parameters on strand dimensions for an extended range of print speed and nozzle gap sizes has been conducted through X-ray micro-computed tomography scanning, aiming to predict geometric characteristics of strand deposition during FFF [33]. Appropriate extrusion parameters were identified for a desired strand geometry, indicating a transition from under-extrusion to over-extrusion.

A few research works have extended the investigation of fundamental FFF structural elements to include both processability and functionality attributes offered by specialty materials, e.g., (nano)composites with enhanced thermal and electrical conductivity. Several applications for FFF demonstrate the integration of electrically conductive structures by using composite materials with different fillers, e.g., carbon nanotubes (CNT), nano-copper wires, carbon black, or graphene [34]. Conductive filaments present several complexities associated with their microstructural compositions and manufacturing processes. From an electrical point of view, their conductivity can be described using the percolation theory that defines, in a stochastic manner, the electric behavior accounting for the creation and destruction of conductive paths [35]. Although the possibility of employing FFF to fabricate structures with embedded sensing elements and functional responses has been explored, few studies derived design principles or rules, while several researchers in this field have highlighted the high electrical resistance values and variability in 3D-printed specimens, with the bond formation and quality identified as root causes for high values of resistance and variability [34,36]. Main outcomes indicate that resistivity can be adjusted by means of the infill orientation as well as the process parameters of extrusion temperature, speed, and flow rate, while, for reaching small resistance values, transitions in the path of the current have to be avoided [34]. The presence of voids reduces the contact of the adjacent strands as well as their effective cross-sectional area and, therefore, may increase resistivity. The conductance within a layer between neighboring strands (intralayer contact resistance) and the conductance via consecutive layers (interlayer contact resistance) were found to contain additional resistance contributions, thus increasing anisotropy in electrical properties [37]. Extrusion width was also found to have a greater influence than layer height [38]. Recently, electrical characterization of single and multiple bond interfaces was performed to understand the scaling law of electrical resistance across bond interfaces. It was found that the interface contributes two-thirds, while the strand bulk contributes one-third, of the measured electrical impedance, with an inversely proportional relation to the extrusion temperature and no significant contribution of printing speed [39]. In addition, a significant increase in resistivity, of three to four times, was found for deposited strands compared with feedstock prior to processing, which was attributed to inherent heterogeneity and multiple zones with different conductivity values distributed over a cross-section of extruded strands. For specimens with printing orientation of 0° and thicknesses of 0.10, 0.19, and 0.33 mm, the resistivity values were 15 ± 0.5, 13 ± 1, and 8.2 ± 0.2 Ohm-cm, respectively; i.e., the thinner the specimens were, the higher their resistivity [40].

For the purpose of contributing to a more comprehensive understanding of bulk material functional properties and their translation in FFF structures, this work aims to provide a methodological framework for the concurrent assessment of the influence of process parameters on FFF strand stability and functional performance. More specifically, printability and functionality attributes related to deviation from nominal geometry and resistivity, respectively, which have been investigated separately in previous studies, are integrated within a systematic analysis of FFF structural elements (1D stacks of FFF strands and 3D blocks). To this end, a new workflow for dimensional deviation analysis is proposed through a comparison of nominal strand geometry with the reconstructed actual geometry obtained via mCT, followed by multivariate and univariate statistical analysis to provide further insight on main effects and interactions on the selected responses.

## 2. Materials and Methods

### 2.1. Materials

For the present investigation, a commercially available conductive polylactic acid (PLA) filament (Conductive PLA, Proto-Pasta, Vancouver, WA, USA) with 1.75 mm diameter was employed as feedstock material. The compound consists of a blend PLA 4043D (Natureworks, Blair, NE, USA) with approximately 25 wt.% conductive carbon black filler [41]. Indicative resistance properties of the compound, according to the producer, are listed below:

- Volume resistivity of injection-molded specimen: 15 ohm-cm.
- Volume resistivity of 3D-printed part along XY plane: 30 ohm-cm.
- Volume resistivity of 3D-printed parts along Z axis: 115 ohm-cm.
- Resistance of a 10 cm length of 1.75 mm filament: 2–3 kohm.

Prior to processing, filament drying was conducted in a hot-air oven at 65 °C overnight.

### 2.2. 3D-Printing Parameters

#### 2.2.1. FFF Process Conditions

Specimens used in this study were produced with a Prusa i3 MK3S+ FFF system equipped with an E3D V6 hot end and 0.4 mm-diameter brass nozzle. Precise leveling and calibration of the FFF system and first-layer settings were conducted prior to each test through an induction auto-leveling sensor (SuperPINDA, Prusa Research, Prague, Czech Republic), and all specimens were printed in the same XY position to prevent leveling variability. Firstly, a preliminary extrudability assessment was conducted to assess flow consistency and extrusion temperature range, and a temperature setpoint of 225 °C (within 220–240 °C reported in the literature) was selected and kept constant for all tests, as partial nozzle clogging was observed occasionally at lower temperatures. To facilitate specimen adhesion, a double-sided textured PEI powder-coated spring steel sheet was employed as printing surface, and bed temperature was maintained at 60 °C during each test. After printing, specimens were left to cool down and carefully detached from the surface. All specimens were gently wiped with ethanol and conditioned prior to testing in a low-humidity enclosure.

#### 2.2.2. Calculation of Volumetric Flow Rate

For the selected nozzle diameter (0.4 mm) and extrusion temperature (225 °C), the maximum volumetric flow rate that can be attained was calculated through a custom gcode script generator, allowing for the extrusion of a predefined amount of material at different filament feed rates [42]. Feed rates corresponding to 2–24 mm^3^/s material volumetric flow rate through the nozzle with a 2 mm^3^/s step increase were tested in triplicates, and extrudates were weighed on a high-precision scale for the comparative assessment of nominal/actual material throughput. Based on the outcomes of this test, volumetric flow rates within 1–4 mm^3^/s range were selected, demonstrating the least deviations among nominal and actual material flow through the nozzle.

#### 2.2.3. Experimental Design

A full factorial experimental design methodology was used to study the main and interaction effects between the selected experimental factors and response variables in 1D stacks of FFF strands. Specifically, two types of responses were experimentally assessed, corresponding to the printability (mean dimensional difference from nominal geometry (DD_mean_) and functionality (resistivity) attributes, respectively. Responses were investigated in relation to three process parameters (factors), as presented in Table 1. Three replicates were assessed for each combination, in accordance with previous studies [36]. The factorial design was generated and analyzed through Minitab (v20.3) statistical software.

**Figure 1:**
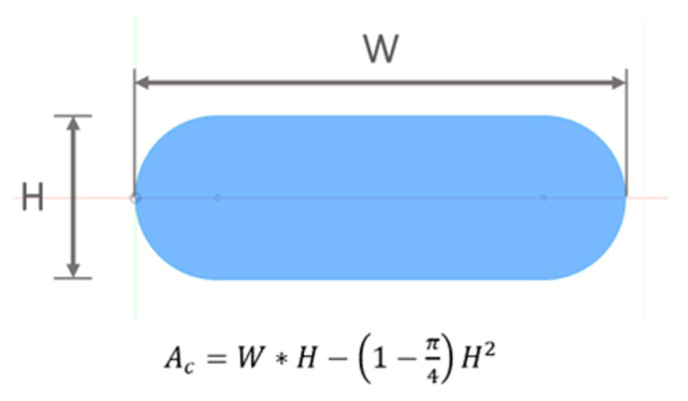
Approximation of the cross-sectional shape of a single FFF strand.

The range of extrusion width (W) and FFF Strand Height (H) parameters is commonly defined as a percentage of nozzle diameter (D_N_). The selection of factors levels was within 20–80% of D_N_ for H and 120–180% of D_N_ for W, respectively, corresponding to the minimum to maximum physical limits of the FFF system (based on preliminary experiments and suggested range by OEM) in order to induce a pronounced effect on selected responses and allow the possibility of assessing printing profiles with different process performance targets, namely, high accuracy/low speed and low accuracy/high speed. Based on the selected levels of W and H parameters, in conjunction with the experimentally defined range of consistent volumetric flow rates (1–4 mm^3^/s), the calculation of the respective feed rate and printing speeds was conducted considering an incompressible, fully developed, laminar flow and applying the mass balance at the outlet of the nozzle, and volumes of deposited FFF strand and material exiting from the nozzle were set as equal according to the volume conservative law. As the volumetric flow rate is directly proportional to the product of the FFF strand cross-sectional area and printing speed, an approximation of the flattened, cross-sectional shape deposited through the nozzle was employed for the calculation of input values E (mm of FFF filament moved towards the liquefier) and F (feed rate in mm/min) required for G-code generation (Figure 1). A cross-sectional shape consisting of two semicircles and a rectangle was selected, commonly employed by slicing software for internal calculations [43]. The list of nominal values of selected experimental factors and calculated FFF strand cross-section areas is presented in Table S1. For each set of parameters, explicit values for E axis and F were calculated for 50 mm length of FFF single strands.

#### 2.2.4. Toolpath Design

**Figure 2:**
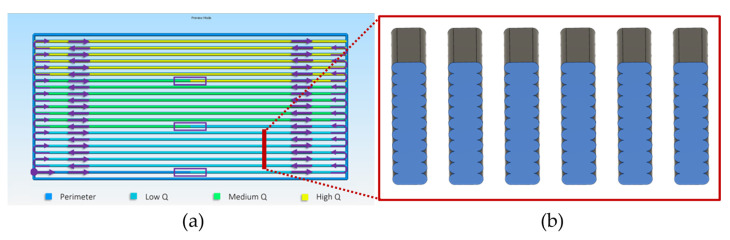
(**a**) Top view of 1D specimens consisting of stacks of single FFF strands with highlighted Q zones and arrows indicating toolpath direction (toolpath visualization with Simplify3D v.4.1.2 software); (**b**) CAD design of Q zone cross-section consisting of six replicates of 1D stacks of FFF strands.

Explicit design of test specimens and printing conditions was conducted with FullControl GCode Designer open-source software (v. Heron02d), allowing one to streamline printing parameters transitions, precisely define toolpath settings, and couple the designed geometry with the manufacturing procedure [44]. Specimens were printed in a uniformly aligned pattern under the DoE-defined printing conditions, consisting of individual 1D stacks of FFF strands grouped in three volumetric flow rate zones with six replicates for each set of parameters, keeping W and H parameters constant, producing a variation of specimens as employed in previous studies (Figure 2) [30].

A perimeter line was introduced for flow stabilization and to facilitate strand attachment during the fabrication process. A transition strand line was employed for transitioning to higher Q values. In order to avoid possible variation introduced from first-layer leveling, constant first-layer settings of 0.5 mm width and 0.2 mm layer height were applied for all specimens printed in the same XY position, thus establishing a common baseline for comparative assessment. Total number of layers was adjusted for each value of H in order to obtain a constant, total height of 2.120 mm for all specimens with 50 mm total length. After fabrication, Q zones were segmented and analyzed with optical microscopy and mCT. For electrical resistance measurements, 1D stacks of FFF strands were carefully detached from the perimeter and measured separately.

**Figure 3:**
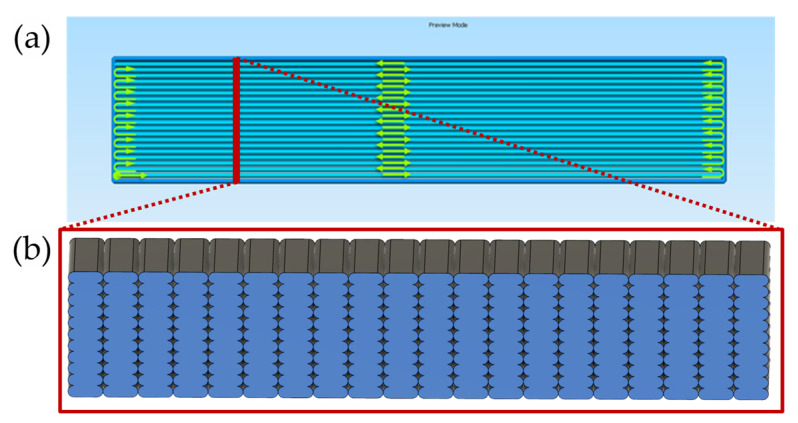
(**a**) Top view of linear 3D blocks (toolpath visualization with Simplify3D 4.1.2 software); (**b**) specimen cross-section consisting of 20 strand stacks overlapping in XY plane.

**Figure 4:**
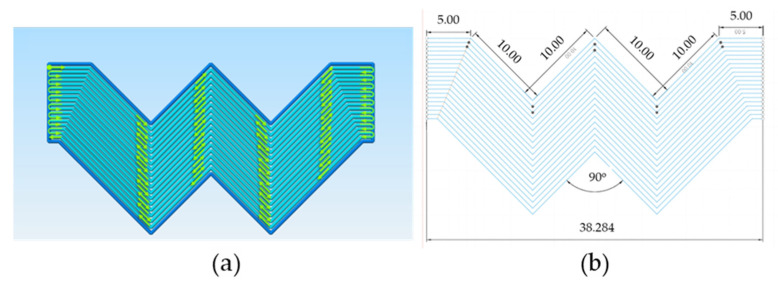
(**a**) Top view of zig-zag 3D blocks; (**b**) trajectory design with constant offset equal to W while maintaining the same active length of 50 mm.

In order to investigate the translation of functional 1D properties in 3D blocks as well as possible effects of change in toolpath orientation in global FFF properties, selected combinations of printing parameters were also investigated in two types of specimens, namely, linear 3D blocks 9.6 × 2.120 × 50 mm (Figure 3) and zig-zag specimens of equal cross-section and active length (Figure 4). In order to avoid overlap of zig-zag toolpath tracks, a constant offset equal to W was applied, and trajectory lines were calculated accordingly (Figure 4b). It is worth noting that the intermediate FFF structural level of 2D layers was not included in the present investigation due to the inherent system limitations in first-layer levelling and challenges in the detachment from the printing surface introducing uncontrolled effects in response variables. As previously specified for 1D analysis, constant first-layer settings of 0.5 mm width and 0.2 mm layer height were also applied for all 3D blocks.

#### 2.2.5. FFF Strand Morphology and Deviation Analysis

**Figure 5:**
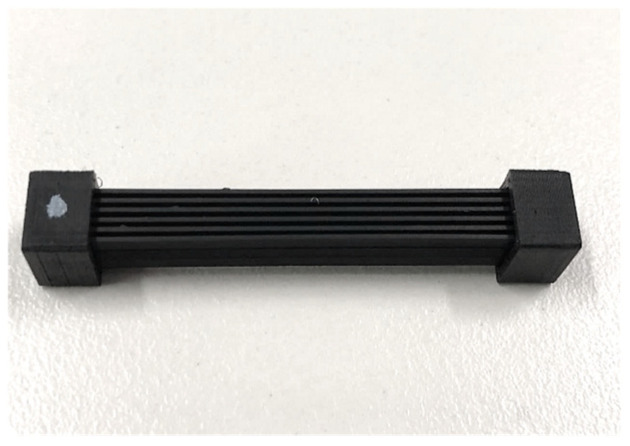
Specimen with 1D stacks of FFF strands prepared for mCT scanning.

The influence of printing parameters on the stability of deposited FFF strands was investigated in terms of dimensional accuracy and surface morphology. Qualitative inspection of top specimen layer (Emspira 3 Digital Microscope, Leica Microsystems GmbH, Wetzlar, Germany) was conducted to assess surface morphology and deposition consistency. Μicro-computed tomography (mCT) was employed for dimensional deviation analysis of 1D stacks of FFF strands through comparison with nominal strand geometry. The principle of mCT relies on accounting for attenuation coefficients of constituent phases (polymer matrix, air) in the beam path. The detected X-rays should be considered with respect to the initial intensity of the X-ray source, according to Beer’s law for expression of linear attenuation. In order to achieve the necessary magnification while maintaining sample volume inside the field of view, specimens produced according to 1D stacks of FFF strands’ toolpath were cut in three segments (low, medium, high Q) and transition lines were removed. A top/bottom rectangle base was employed for segment alignment and positioning on the sample holder (Figure 5).

The samples were scanned with SkyScan 1272 High-Resolution Micro-CT (Bruker micro-CT, Kontich, Belgium), which employs a cone-shaped tomographic imaging beam with a rotating sample holder. During image acquisition, the samples rotated over 360 degrees with a fixed rotation step. At each angular position, a shadow (transmission) image was acquired. The selected acquisition settings are presented in Table 2.

The obtained shadow angular projections were used for the reconstruction of the virtual slices through each sample. Grayscale cross-sections were generated using NRecon reconstruction software (v1.7.0.4 by Bruker micro-CT) by implementing the Feldkamp algorithm. The lower limit of the dynamic image range was fixed at 0 (corresponding to air) and upper limit was selected based on sample contrast.

**Figure 6:**
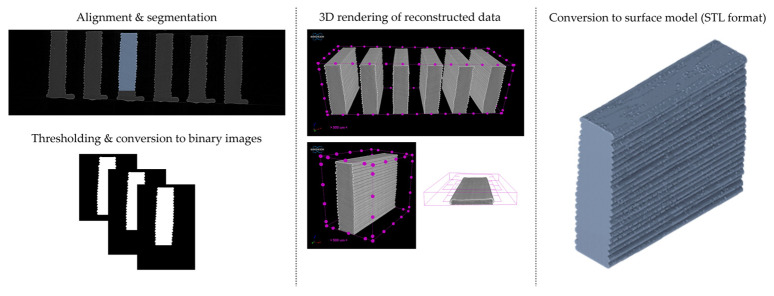
Digital workflow for the generation of reconstructed 3D surface models of actual geometry (1D stacks of FFF strands).

Dimensional deviation analysis was conducted through a comparison of the 3D surface model of nominal stack geometry with the reconstructed 3D surface model of actual geometry for each set of parameters. Datasets collected from each 1D stack (same printing parameters) were analyzed as individual replicates of the experiment. The 3D surface model of nominal stack geometry was designed in Autodesk Fusion 360 CAD software (v.16.3.0.2035) and exported as an STL file. For the reconstructed 3D model of actual geometry, Otsu’s method for automatic image thresholding was applied on the original grayscale slices, followed by a marching cubes high-resolution 3D surface construction algorithm in CT-Analyser software (v1.18.4 by Bruker micro-CT) (Figure 6). In order to reduce uncontrolled effects from first-layer levelling, the bottom three layers of each replicate were excluded from the analysis.

**Figure 7:**
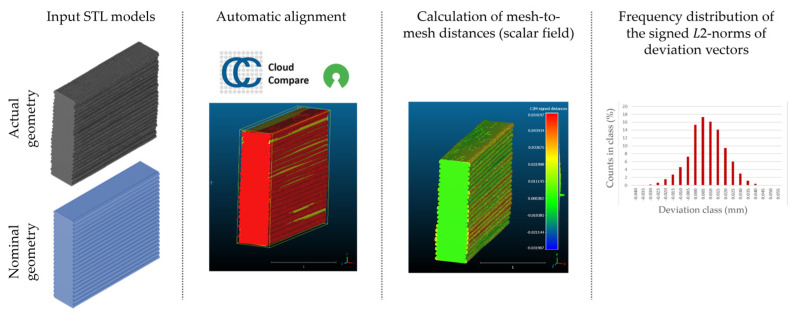
Nominal-to-actual STL alignment for dimensional deviation analysis in CloudCompare (v2.13) software. The minimum RMS improvement was set at 10^−5^ between consecutive iterations.

Subsequently, nominal to actual STL alignment (registration) was conducted in CloudCompare open-source software (v2.13), employing the iterative closest point (ICP) algorithm to iteratively calculate the rigid body transformation (translation and rotation) that minimizes the mean of squared distances (RMS) between the corresponding pairs of STL-CAD vertices (Figure 7).

Once the scan data and CAD reference were properly aligned, the signed L2 norms of deviation vectors from CAD to scan were calculated, where positive values indicate a positive deviation (over-extrusion) and negative values indicate under-extrusion, respectively. The obtained values of signed L2 norms were then classified in deviation classes (0.005 mm-class range), to obtain the frequency distribution for each replicate, along with descriptive statistics to analyze central tendency and variability.

**Figure 8:**
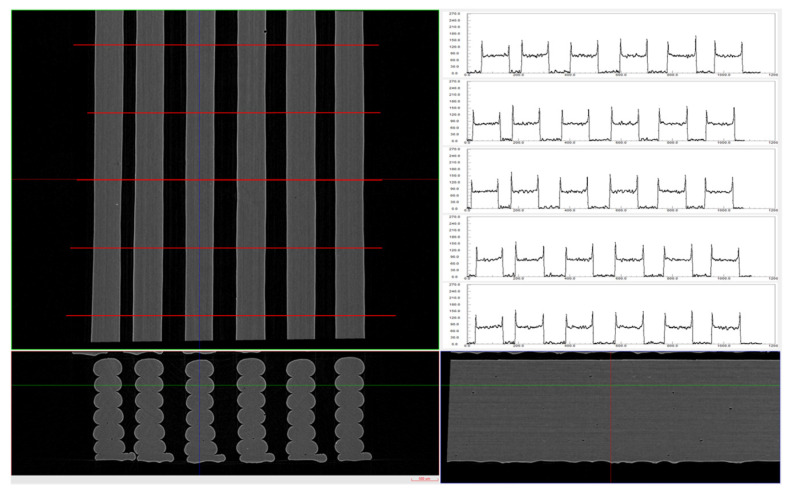
XZ, XY, and ZY cross-sections (**left**) and respective profile lines (**right**) perpendicular to printing direction for calculation of average extrusion width (DataViewer software v.1.2.5.7, Bruker micro-CT). Cross-section image scale: 500 um.

Given the restriction of strand height between the nozzle tip and preceding layer, extrusion width variability is affected by the FFF process parameters more directly. Complementary to dimensional deviation analysis (global analysis), explicit measurements of extrusion width were also carried out locally as a means for further comparison. To this end, the original grayscale slices were processed in DataViewer software (version 1.2.5.7 by Bruker micro-CT) to align and extract profile lines across single FFF strands perpendicular to the printing direction (Figure 8). Profile data files in .csv format were subsequently postprocessed with a custom python script, to calculate extrusion width values and actual-to-nominal extrusion width ratio, and grouped for each stack across all layers of interest.

#### 2.2.6. Resistance Measurements

**Figure 9:**
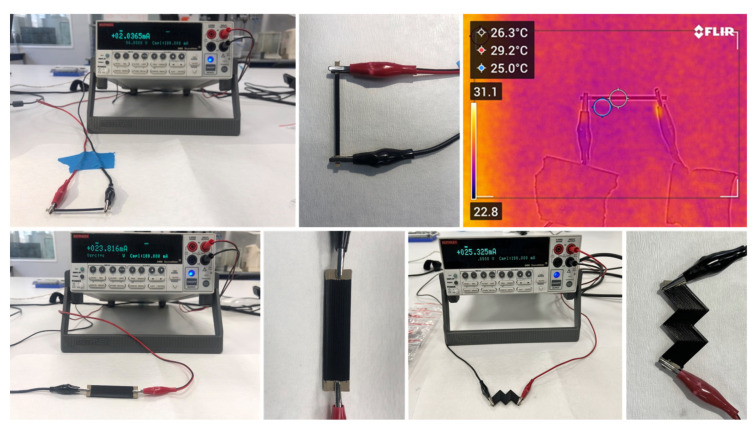
Experimental setup for two-point probe resistivity measurement and specimen temperature monitoring with IR camera.

The electrical resistance measurements were made along the length of the specimens using a two-point probe configuration in which electrical contacts were established with silver paint. This method was previously reported in the literature to demonstrate low bonding resistance and ease of application over other variants of electrical bonding [34,40]. Two contact points at 40 mm distance (active length) were applied for measuring specimen resistance. A source measure unit instrument (2400 Series SourceMeter^®^ Lines, Keithley Instruments Inc., Cleveland, OH, USA) was employed for measuring direct current (DC) resistance; this instrument was controlled through a user interface developed in LabVIEW^®^ (by National Instruments Corp. Austin, TX, USA) [45], which provided real-time information about the system’s state and storage of data for postprocessing (Figure 9). Constant voltage values of 1.5–3.0–4.5–6.0 V (relevant to low-voltage applications) were applied, and resistance and current output values were recorded over a period of 240 s. During each measurement, specimen temperature was monitored through an IR camera (FLIR C5, Teledyne FLIR, Wilsonville, OR, USA). For the selected low voltage range, no increase in specimen temperature was observed.

**Figure 10:**
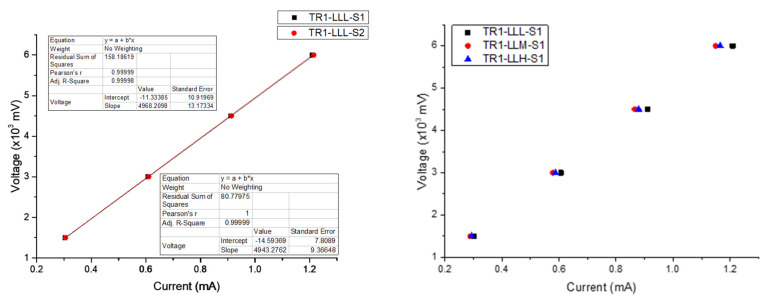
Indicative voltage versus current plots demonstrating highly repeatable ohmic response. (**left**): samples obtained from the same process parameters; (**right**): samples with equal W, H, and increasing Q.

Volume resistivity values were calculated from the measured resistance and specific dimensions of each specimen’s geometry. In order to measure the resistivity, the ohmic response of measured samples was confirmed within the selected 1.5–6.0 V range, without any measurable contribution of capacitive effects (Figure 10).

The electrical volume resistivity of the samples was therefore evaluated by Equation (1):

(1) $$\rho =R*\frac{A_{c}}{L}$$

where *R* is the measured resistance (Ohm), *A_c_* the actual cross-section of 1D stacks of FFF strands (mm^2^) measured via mCT, and *L* is the defined active length between contact points, equal to 40 mm.

#### 2.2.7. Statistical Analysis

Minitab software (v20.3) was used to perform multivariate and univariate analysis of variance (ANOVA). The strength of association between the selected response variables was assessed by Pearson’s correlation coefficient. The null hypothesis of homoscedasticity for response variables versus H, W, and Q was confirmed with Levene’s test. Multivariate ANOVA was used to determine whether there are significant differences across factor levels for the combination of correlated response variables. The analysis was conducted based on Wilk’s lambda F-values and *p*-values to determine the significance of main effects and interaction terms. In order to provide further insight into individual response variables that drive the significant multivariate effects, univariate ANOVA statistical tests were conducted on each response. Statistically significant terms (*p*-value ≤ 0.05) in the ANOVA results were identified to interpret the effects of the process parameters on dimensional accuracy and functionality performance.

## 3. Results and Discussion

### 3.1. Calculation of Volumetric Flow Rate

A predefined amount of material corresponding to 200 mm of filament length was extruded at different feed rates. The obtained extrudate weights for each nominal flow rate are presented in Table S2. In order to assess under-extrusion percentage and actual volumetric flow rate, all values were normalized with the average weight of 2 mm^3^/s extrudates, assuming the actual and nominal volumetric flow rate are equal at this value, as it corresponds to minimum shear stresses applied during nozzle extrusion. Based on the results obtained, volumetric flow rates within the 1–4 mm^3^/s range were selected for subsequent tests, safely within the acceptable window with non-significant material underflow.

### 3.2. 3D Strand Analysis

#### 3.2.1. Surface Morphology

The effect of strand height versus extrusion width, in terms of top surface morphology and deposition consistency, can be observed in Table 3, indicatively presented for the same value of volumetric flow rate of 4 mm^3^/s. Qualitative inspection of the specimens’ top view was conducted near the center of total strand length, where the targeted printing speed has been reached and no influence from acceleration is foreseen. The flattened area resulting from nozzle tip compression during strand deposition is indicative of the active welding area between adjacent strands in Z direction. As expected, increasing nominal extrusion width promotes the active welding area. For the same value of W, the effect of nominal strand height increase is demonstrated through a transition from a compressed strand morphology towards a more circular shape with reduced active welding area. Some flow inconsistencies and fluctuation can be observed in marginal top H and W values (TR8-MHH, TR9-HHH), indicative of physical limitations of material flow and spreading imposed on the actual strand geometry that can be obtained. The effect of extrusion width versus volumetric flow rate can be observed in Table 4, indicatively presented for the same value of strand height of 0.32 mm.

An increase in deposition consistency and reduced surface texture is observed at higher volumetric flow rates. Similar surface defects have been previously reported in the literature for low printing speeds, associated with insufficient extrusion pressure and upward shift of the solid–liquid interface in the nozzle, allowing bubbles to propagate [46]. However, as the localization of defects was observed near the strand surface and not in the main strand volume (Figure S1), potential root causes may rely on the reduction of residence time of melted material inside the liquefier for higher printing speeds, associated with reduced thermal loading, or wall slip phenomena during extrusion. The influence of humidity is not considered significant, given that the same drying and storage protocol was followed for all experiments.

#### 3.2.2. Dimensional Deviation Analysis

The signed L2 norms of deviation vectors from CAD to scan were calculated for three replicates of each experimental run. The frequency distribution for each replicate was obtained by classifying the signed L2 norms in 35 deviation classes in the range of −0.085 to 0.085 mm, listing the number of counts of deviations within each class expressed as a percentage over the total number of counts. For the visualization of the deviation vector field, colormaps with respect to the original CAD model were generated. The sign of L2 norms represents the direction of the deviation; namely a negative value (towards the blue color range) indicates that the deviation vectors point towards the inside of CAD reference geometry and vice versa. Colormaps and frequency distributions of signed L2 norms of deviations are presented in Table 5 for different sets of process parameters.

As observed in the reconstructed 3D models of actual strand geometry presented in Table 5, and in accordance with other studies, layer height greatly influences the cross-sectional geometry of extruded strands [43,47,48]. At the low layer height values, the space between the nozzle and preceding layer is relatively limited compared to the volume of extruded material. As a result, the molten material is pressed and forced to spread to the sides, resulting in high aspect ratio cross-sections. The flat-top shapes of the extruded strands are commonly observed at low layer height values, and a transition towards more semi-circular shapes can be seen at increased layer heights. With respect to the frequency distributions of signed L2 norms of deviations, a distinct footprint of process parameters on central tendency and variability is observed. A desirable distribution form would consist of a narrow peak centered around zero, corresponding to high accuracy and precision [49]. Respectively, the presence of over/under-extrusion is demonstrated by positive/negative skewness and bimodal patterns, while a broadened distribution is associated with strand deposition inconsistencies.

For the comparative assessment of dimensional accuracy and precision, the arithmetic mean of dimensional deviations over all classes (DD_mean_) and its standard deviation (SD_DD_) were calculated. In addition, the arithmetic mean of absolute dimensional deviations (|DD|_mean_) as well as mean values of positive and negative range (DD_+_ and DD_−_, respectively) were calculated to provide insight into the contribution of over/under-extrusion. Complementary to dimensional deviation analysis (global analysis), explicit measurements of extrusion width were also carried out locally, and the ratio of actual extrusion width (arithmetic mean) over nominal width was calculated as a means for further comparison (W_m_/W_n_). The results of dimensional accuracy measurements for 1D FFF strands are summarized in Table 6, where a color scale was applied to indicate where each value falls within the max/min column range. The largest deviations, in terms of over-extrusion, are observed for TR3-HL, TR6-HM, and TR9-HH, obtained from the high level of strand height (0.32 mm). A less pronounced effect is observed for extrusion width, e.g., for TR1-LL and TR4-LM; however, in the case of TR7-LH, equally large contribution from DD_+_ and DD_−_ is indicative of strand deposition inconsistency at the combination of low H (0.32 mm) with high W (0.72 mm). Finally, improved precision and accuracy are observed for TR1-LL, TR4-LM, and TR5-MM. The values obtained for DD_mean_, encompassing both over- and under-extrusion contributions, were selected as a global response of dimensional accuracy and precision for the subsequent statistical analysis.

#### 3.2.3. Resistance Measurements

**Figure 11:**
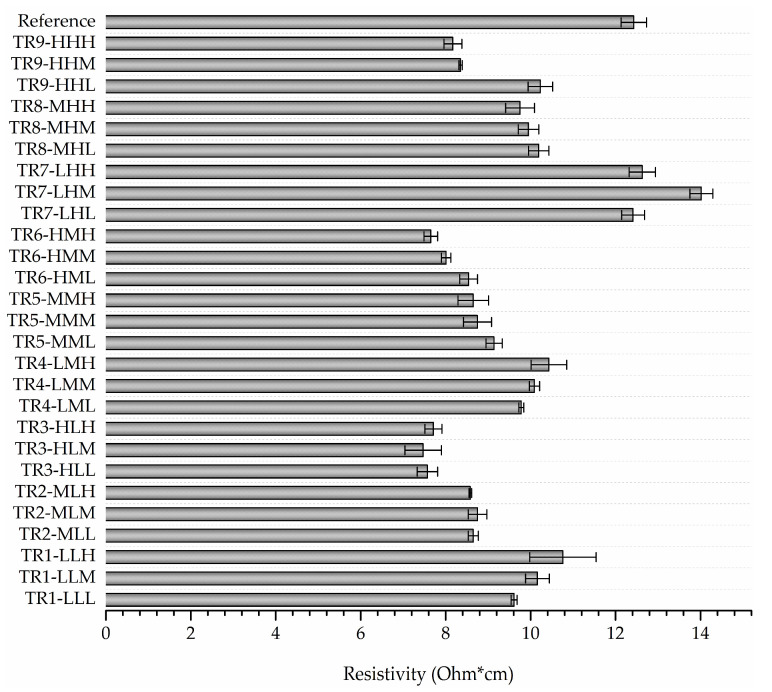
Resistivity response for the investigated combinations of process parameters.

The measured resistance and cross-section values (derived from mCT analysis) were employed for the calculation of resistivity response, as presented in Figure 11 and Table S3. A notable decrease in resistivity is observed with increasing layer height (TR3-HL, TR6-HM, and TR9-HH), with the lowest resistivity values obtained for TR3-HL and the highest values for TR7-LH. As the increase in the total number of layers is associated with smaller values of strand height, this variation in resistivity response is associated with additional contributions from interlayer resistance due to transitions between subsequent strands in Z direction [37,43]. In addition, in the case of TR7-LH, as negative values of DD_mean_ are related to material under-extrusion, the respective reduction of active bonding areas between adjacent FFF strands is also reflected in larger resistivity values. By comparing the obtained resistivity values of single strands with the resistivity of the filament feedstock prior to processing (reference), all parameter combinations exhibit lower resistivity up to 38% (in the case of TR6-HMH), with the exception of TR7 variants. Contrarily, resistivity results reported by other studies demonstrated that lower FFF structural levels led to an increase in resistivity compared to the initial filament; however, the presence of distinct zones inside the specimens was also identified, which could hypothetically explain resistivity alterations of the filament during strand deposition [40]. In addition, lower speeds have been reported to result in lower resistivity due to a higher energy input and increased accuracy [34]; however, no specific pattern can be observed for stacked 1D strands included in this investigation.

#### 3.2.4. Statistical Analysis

**Figure 12:**
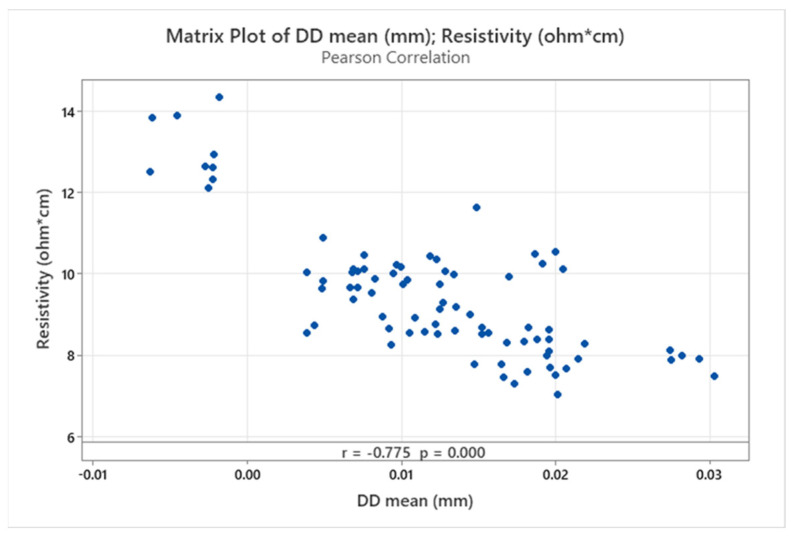
Scatter plot matrix and Pearson’s correlation coefficients between response variables.

The strength of association between the two response variables (DD_mean_ and ρ) was assessed by Pearson’s correlation coefficient. A statistically significant correlation of −0.775 was calculated (*p* value < 0.001), indicating a large negative correlation between the two responses (Figure 12).

As described in the previous section, this correlation is potentially associated with additional contribution from interlayer resistance, due to a higher number of transitions between subsequent strands for low H values, as well as reduction of active bonding areas between adjacent FFF strands in the case of under-extrusion. Correspondingly, resistivity values gradually decrease with increasing DD_mean_, as over-extrusion promotes neck formation and welding between adjacent strands at the cost of dimensional accuracy.

The null hypothesis of homoscedasticity for D_mean_ versus H, W, and Q and for ρ versus H, W, and Q was confirmed with Levene’s test *p*-values of 0.812 and 0.773, respectively, greater than the common significance level of 0.05. Multivariate ANOVA was used to determine whether there are significant differences across factor levels for the combination of response variables. The analysis was conducted based on Wilk’s lambda F-values and *p*-values, as presented in Table 7, to determine the significance of main effects and interaction terms. Multivariate ANOVA calculations showcase that each input variable (H, W, Q) and interaction term (H:W, H:Q, W:Q) is statistically significant. Based on the computed F-values, the main effects of factors H and W as well as the H:W interaction term have the greatest influence in dimensional accuracy and resistivity. Despite the fact that all remaining effects and interactions (Q, H:Q, and W:Q) are also statistically significant (*p* < 0.05), their respective F-values are noticeably lower than the aforementioned. The latter leads to the conclusion that H and W play a pivotal role in both printability and functionality of FFF strands.

**Figure 13:**
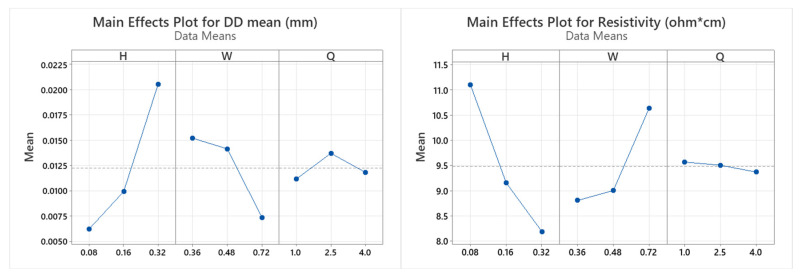
Main effects plot for D_mean_ and ρ responses versus H, W, and Q factors.

In order to provide further insight into individual response variables that are driving the significant multivariate effects, univariate ANOVA statistical tests were conducted on each response (D_mean_ versus H, W, and Q and ρ versus H, W, and Q). As previously, a *p*-value of less than 0.05 was selected to represent a significant effect. The results of the univariate ANOVA are summarized in Table 8. By comparing results obtained from multivariate and univariate ANOVA, alignment on the major significance of main effects of H and W factors and H:W interaction is confirmed for both D_mean_ and ρ. Specifically, FFF strand height has the greatest influence on strand dimensional accuracy (printability attribute) and resistivity (functionality attribute). In addition, the contribution of volumetric flow rate in resistivity response is not statistically significant. As can be observed by the main effects plots for D_mean_ and ρ (Figure 13), the trade-off between the two responses can be obtained near the medium range of H and W values, as a respective reduction of resistivity is attained by reducing extrusion width and increasing strand height at the cost of dimensional accuracy (D_mean_ increase), as previously identified by the negative correlation of the two response variables.

**Figure 14:**
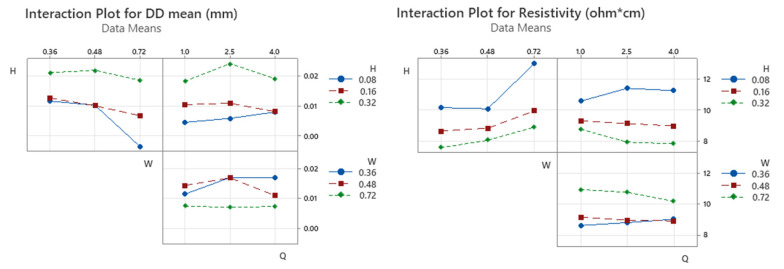
Interaction plots for D_mean_ and ρ responses versus H, W, and Q factors.

Figure 14 shows that the interaction between strand height and extrusion width is more pronounced at combinations of min/max factor levels, i.e., H0.08 × W0.72 mm and H0.32 × W0.36 mm, without notable contribution from the level of volumetric flow rate. This interaction is associated with the distinct differences in the cross-sectional geometry of the extruded strands, indicative of how the print parameters influence strand geometry, such that the basic building blocks of the FFF process can be locally manipulated and controlled. Values of 0.08–0.16 mm for strand height maintain a stable response, with increasing extrusion width within the 0.36–0.48 mm range.

Conclusively, while multivariate ANOVA acknowledges all factor correlation scenarios as statistically significant, the univariate ANOVA statistical tests dedicated to each response exclusively indicate that the contribution of volumetric flow rate in resistivity response is not statistically significant. These findings are in line with the framework of each analysis, since the first pathway investigates the relationship between both response variables with regard to employed factors, while the latter deploys analysis scenarios exclusively for each response variable. The results obtained from the full factorial design analysis aim to provide a common basis for future evaluation of the modelling and predictive efficiency of fractional factorial design models and statistical tools, e.g., Taguchi and response surface experimental designs, for the reduction of experimental runs.

##### 3.3. 3D Block Analysis

The translation of bulk material functionality (resistivity) in the respective FFF counterparts was assessed in a subsequent analysis of 3D blocks (Table 9). Following the obtained outputs and aiming to streamline experiments for the assessment of 3D blocks, candidate factor combinations were selected based on the results of the analysis of the 1D stacked FFF strands. Given that strand height had the most significant influence on both response variables, this factor was selected to be investigated in three levels, as previously, and two toolpath configurations (linear and zig-zag geometry), keeping W and Q constant. Medium level values of 0.48 mm and 2.5 mm^3^/s for extrusion speed and volumetric flow rate were selected for this analysis.

###### 3.3.1. Surface Morphology

The effect of layer height versus toolpath design, in terms of top surface morphology and deposition consistency, can be observed in Table 10. For specimens fabricated with a linear toolpath, qualitative inspection was conducted near the center of the specimen, while in the case of a zig-zag toolpath, areas of 90° shift of toolpath orientation were assessed, subjected to acceleration during deposition. In the case of 3D-TR4-LMM (0.08 mm layer height), increased welding between XY adjacent strands can be observed due to the high degree of strand compression, with a common pattern of marginally visible weld lines among three consecutive strands. Respectively, an intermediate effect is observed for 3D-TR5-MMM (0.16 mm layer height), where strand edges are more visible, and a more uneven surface morphology is observed. This effect becomes more pronounced for the highest value of layer height (0.32 mm), where visible effects of material overflow and increased surface texture are observed. In terms of acceleration effects, the shift of toolpath orientation introduces lingering vibrations due to the inertia of the printing head; these vibrations are more evident at higher printing speeds (TR4, 68 mm/s) and become less evident at lower speeds (TR6, 19 mm/s) for the same value of volumetric flow rate (2.5 mm^3^/s). In addition, distinct surface texture characteristics observed in 1D strands seem to be alleviated and homogenized by multiple XY passes of the printhead nozzle.

###### 3.3.2. Resistance Measurements

**Figure 15:**
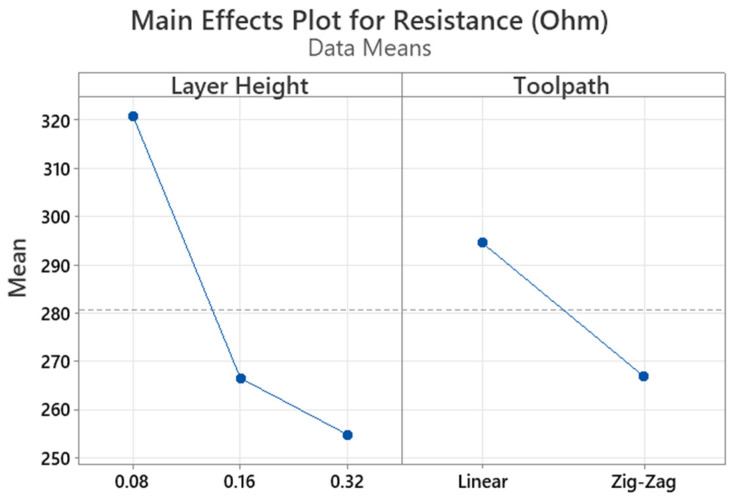
Main effects plot for R response versus LH and TP factors.

An increase in resistivity response was recorded for 3D blocks, in comparison with stacked 1D strands, as observed in resistance and associated resistivity values presented in Table 11. By comparing resistivity values among linear toolpath specimens and reference material prior to processing, higher resistance is observed for 3D blocks. Although a common trend is observed for the effect of layer height between stacked 1D strands and 3D blocks, with the lowest resistivity values obtained for 3D-TR6-HMM, the overall effect of process parameters is less pronounced than in the case of 1D stacks. It is thus possible the translation of 1D strands’ properties to 3D blocks results in a trade-off among competing mechanisms, i.e., the formation of a 3D network of conductive pathways that facilitates current flow, the associated increase in inter/intra-layer contact resistance among multiple strands, and the higher probability of structural defects being present in the 3D structures that may hinder current flow. Nonetheless, the majority of values obtained are within the resistivity range provided by the material producer for injection-molded specimens (15 ohm-cm) and lower than the resistivity of the 3D-printed part along the XY plane (30 ohm-cm). Also, by comparing resistivity values obtained from different toolpath designs (linear and zig zag), a reduction of approximately 15% is observed for zig-zag specimens, with the assumption that nominal active lengths remain equal (40 mm). However, in the case of zig-zag toolpaths, the definition of the explicit value of active length is non-trivial, as interlayer pathways are created across the XY plane that possibly reduce the actual active length between probe contact points. The subsequent statistical analysis was thus conducted for resistance values, previously reported as an alternative response for comparative assessment for the same material volume and specimen cross-section [36]. Both layer height and toolpath design factors have statistically significant main effects on resistance response, as indicated by Table 12 and Figure 15, with layer height having a great influence (F = 77.56), followed by toolpath design (F = 35.94), while a non-significant interaction between the two factors is observed.

## 4. Conclusions

The present study proposes a methodological framework for the concurrent assessment of the influence of process parameters on FFF strand stability and functional performance through a systematic analysis of FFF structural elements (1D stacks of FFF strands and 3D blocks). The selection of factor levels was within minimum/maximum physical limits of the FFF system, namely, 0.08–0.32 mm for H and 0.36–0.72 mm for W, respectively, and printing speed was selected in accordance with the actual volumetric flow rate that can be attained (1–4 mm^3^/s). A full factorial experimental design methodology was used to study the main and interaction effects, aiming to provide a common basis for future evaluation of the modelling and predictive efficiency of fractional factorial design models and statistical tools, e.g., Taguchi and response surface experimental designs, for the reduction of experimental runs. Explicit design of test specimens and printing conditions was conducted, allowing for the streamlining of printing parameter transitions, precisely defining toolpath settings, and coupling the designed geometry with the manufacturing procedure, thus reducing the possible influence of slicing software.

Through the qualitative inspection of 1D stacks of FFF strands with optical microscopy, the increase in nominal extrusion width was observed to promote the active welding area. For the same value of width, the effect of nominal strand height was demonstrated through a transition from a compressed strand morphology towards a more circular shape with reduced active welding area. Some flow inconsistencies and fluctuation were observed in marginal top values (H0.16:W0.72:Q4 and H0.32:W0.72:Q4), indicative of physical limitations of material flow and spreading, imposed on the actual strand geometry that can be obtained. In 3D blocks, the distinct surface texture characteristics observed in 1D strands seem to be alleviated and homogenized by multiple XY passes of the printhead nozzle, although visible effects of material overflow and increased surface texture were observed for the highest value of layer height (0.32 mm).

For dimensional deviation analysis, a new workflow is proposed through the comparison of nominal strand geometry with the reconstructed actual geometry obtained via mCT. The signed L2 norms of deviation vectors from CAD to scan were calculated and classified in deviation classes to obtain the frequency distribution for each replicate, with a distinct footprint of process parameters observed on central tendency and variability. The largest deviations, in terms of over-extrusion, were observed for the high level of strand height (0.32 mm), and a less pronounced effect was observed for extrusion width within 0.36–0.48mm range. Finally, improved precision and accuracy were obtained for H0.08W0.36, H0.16W0.48, and H0.16W0.48 combinations.

Finally, a negative correlation between dimensional accuracy and resistivity was found in 1D stacks of FFF strands, with variants of 0.32 mm strand height exhibiting low resistivity and high dimensional deviations. This effect is potentially associated with additional contribution from interlayer resistance due to a higher number of transitions between subsequent strands for low H values as well as reduction of active bonding areas between adjacent FFF strands in case of under-extrusion. In accordance with previous studies, FFF strand height had the greatest influence on dimensional accuracy (printability attribute) and resistivity (functionality attribute). In addition, the contribution of volumetric flow rate and associated printing speed in resistivity response was not found to be as significant. The trade-off between the two responses was obtained near the medium range, with values of 0.16 mm for strand height maintaining a stable response with increasing extrusion width within the 0.36–0.48 mm range. Although a common trend was observed for the effect of layer height between stacked 1D strands and 3D blocks, with the lowest resistivity values obtained for H0.32W0.48, the overall effect of process parameters is less pronounced than in the case of 1D stacks. It is thus possible that the translation of 1D strands properties to 3D blocks results in a trade-off among competing mechanisms, i.e., the formation of a 3D network of conductive pathways that facilitates current flow, the associated increase in inter/intra-layer contact resistance among multiple strands, and the higher probability of structural defects being present in the 3D structures that may hinder current flow. Both layer height and toolpath design factors were found to have statistically significant main effects on resistance response. Although focus was given on in-plane resistivity measurement in order to rationalize the total number of experimental runs, the developed methodology can be easily extended to assess cross-plane resistivity and anisotropic effects.

The proposed framework enables the quantification of the influence of process parameters on the selected response variables, contributing to the development of standard operating procedures and recommendations for selecting optimal process parameters to achieve the desired process stability and functional performance in FFF. Main effects and interactions were associated with the distinct differences in cross-sectional geometry of the extruded strands, indicative of how the print parameters influence strand geometry such that the basic building blocks of the FFF process can be locally manipulated and controlled. The main outcomes will further enable informed design decisions and modeling of FFF electronics and sensors where different process parameter sets may be required to be applied according to geometrical constraints or process accuracy/time optimization goals.

## Figures and Tables

**Table 1:** Overview of experimental factors, control levels, and response variables.

Control Factor	Factor Ref.	Units	Levels			Response Variables
			−1	0	1	
FFF strand height	A	mm	0.08	0.16	0.32	- Mean dimensional Difference (DD_mean_) - Resistivity (ρ)
Extrusion width	B	mm	0.36	0.48	0.72	
Volumetric flow rate	C	mm^3^/s	1	2.5	4	

**Table 2:** Acquisition settings for mCT scanning.

Filter = no filter	Exposure (ms) = 650
Source voltage (kV) = 50	Rotation step (deg) = 0.150
Source current (uA) = 180	Frame averaging = ON (3)
Image pixel size (um) = 5	Random movement = OFF
Camera binning = 2 × 2	Flat-field correction = ON
Reference intensity = 57,000	Scan duration = 2 h

**Table 3:** Top view of FFF strand morphology with increasing H and W, indicatively presented for Q = 4 mm^3^/s (scale bar = 500 um, identical for all images).

	Increasing H	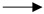	
Increasing W	TR1-LLH	TR2-MLH	TR3-HLH
	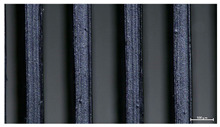	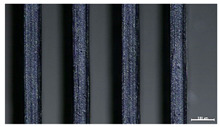	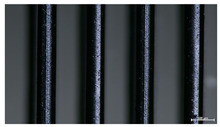
	TR4-LMH	TR5-MMH	TR6-HMH
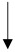	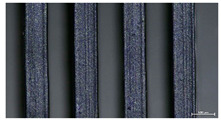	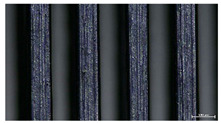	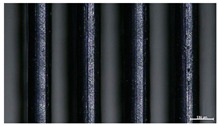
	TR7-LHH	TR8-MHH	TR9-HHH
	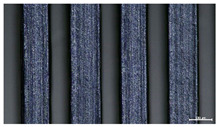	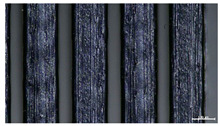	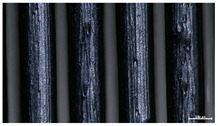

**Table 4:** Top view of FFF strand morphology with increasing W and Q, indicatively presented for H = 0.32 mm (scale bar = 500 um, identical for all images).

		Increasing Q	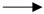	
		Q = 1 mm^3^/s	Q = 2 mm^3^/s	Q = 4 mm^3^/s
Increasing W	TR3-HL	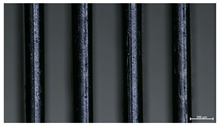	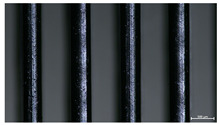	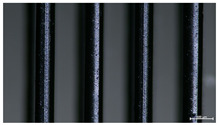
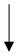	TR6-HM	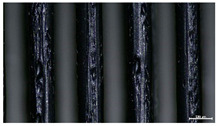	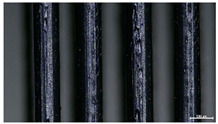	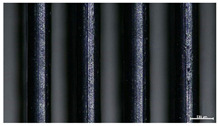
	TR9-HH	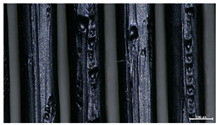	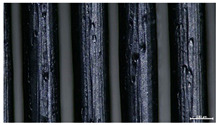	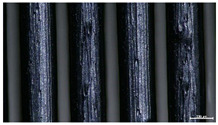

**Table 5:** **Left**: indicative colormaps of signed L2 norms of deviation vectors, where negative values (towards blue color range) correspond to directions towards the inside of CAD reference geometry (scale bar = 1 mm, identical for all images). **Right**: frequency distribution of classified, signed L2 norms of deviation vectors.

TR1-LL	** 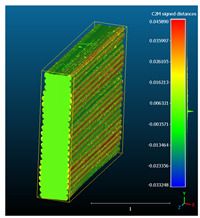 **	** 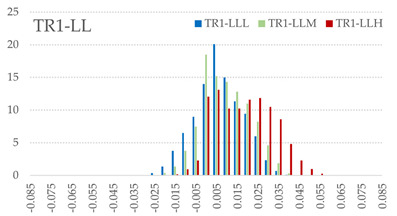 **	
TR2-ML	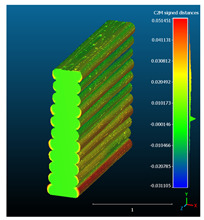	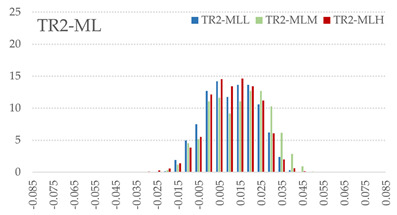	
TR3-HL	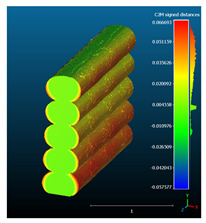	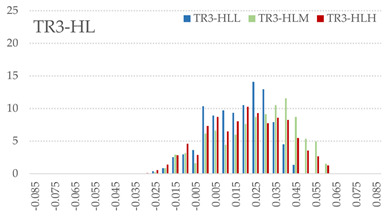	
TR4-LM	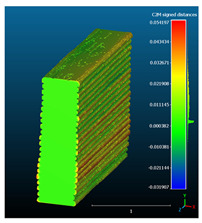	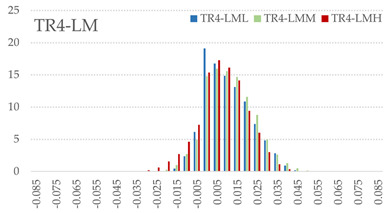	
TR5-MM	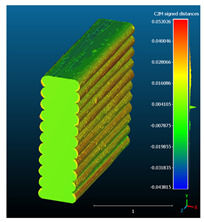	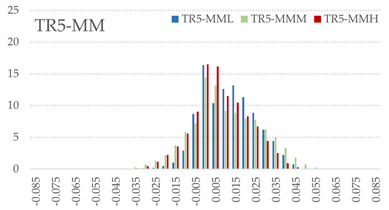	
TR6-HM	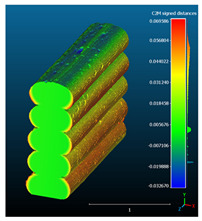	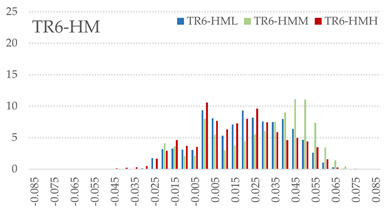	
TR7-LH	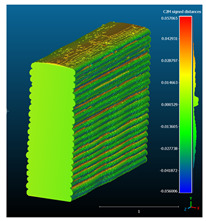	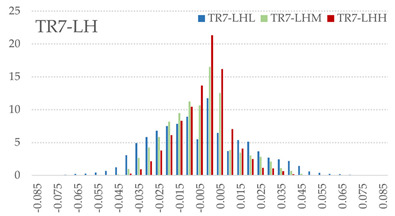	
TR8-MH	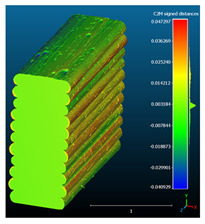	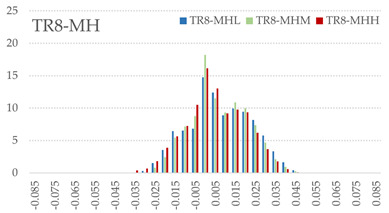	
TR9-HH	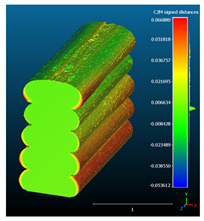	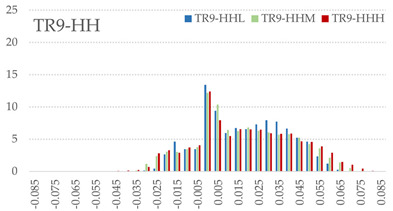	

**Table 6:** Calculated mean values of dimensional deviations for 1D FFF strands (total average values for 3 replicates); DD_mean_: mean of dimensional deviations over all classes; SD_DD_: standard deviation of DD_mean_, |DD|_mean_: arithmetic mean of absolute dimensional deviations; DD_+_ and DD_−_: mean values of positive and negative range; W_m_/W_n_: ratio of actual extrusion width (arithmetic mean) over nominal width.

	A	B	C	DD_mean_ (mm)	SD_DD_ (mm)	|DD|_mean_ (mm)	DD_−_ (mm)	DD_+_ (mm)	W_m_/W_n_
TR1-LLL	−1	−1	−1	0.007	0.012	0.011	−0.010	0.013	1.010
TR1-LLM	−1	−1	0	0.010	0.011	0.012	−0.008	0.015	1.027
TR1-LLH	−1	−1	1	0.018	0.014	0.019	−0.007	0.021	1.097
TR2-MLL	0	−1	−1	0.011	0.012	0.014	−0.008	0.017	1.016
TR2-MLM	0	−1	0	0.015	0.014	0.017	−0.008	0.020	1.066
TR2-MLH	0	−1	1	0.012	0.012	0.014	−0.009	0.017	1.038
TR3-HLL	1	−1	−1	0.017	0.015	0.019	−0.011	0.022	1.115
TR3-HLM	1	−1	0	0.026	0.019	0.028	−0.012	0.031	1.164
TR3-HLH	1	−1	1	0.021	0.019	0.024	−0.012	0.027	1.160
TR4-LML	−1	0	−1	0.011	0.011	0.012	−0.007	0.016	1.005
TR4-LMM	−1	0	0	0.012	0.012	0.013	−0.008	0.016	1.027
TR4-LMH	−1	0	1	0.008	0.011	0.012	−0.010	0.014	1.030
TR5-MML	0	0	−1	0.012	0.013	0.014	−0.008	0.018	1.056
TR5-MMM	0	0	0	0.011	0.016	0.016	−0.012	0.019	1.014
TR5-MMH	0	0	1	0.008	0.014	0.012	−0.011	0.015	1.020
TR6-HML	1	0	−1	0.020	0.021	0.024	−0.014	0.028	1.110
TR6-HMM	1	0	0	0.028	0.023	0.032	−0.013	0.036	1.147
TR6-HMH	1	0	1	0.018	0.022	0.023	−0.015	0.027	1.092
TR7-LHL	−1	1	−1	−0.004	0.024	0.020	−0.022	0.020	0.802
TR7-LHM	−1	1	0	−0.004	0.016	0.013	−0.017	0.013	0.869
TR7-LHH	−1	1	1	−0.002	0.013	0.010	−0.013	0.011	0.928
TR8-MHL	0	1	−1	0.008	0.016	0.014	−0.012	0.018	1.054
TR8-MHM	0	1	0	0.007	0.014	0.012	−0.011	0.017	1.044
TR8-MHH	0	1	1	0.005	0.015	0.012	−0.012	0.015	1.032
TR9-HHL	1	1	−1	0.018	0.021	0.022	−0.012	0.028	1.094
TR9-HHM	1	1	0	0.018	0.023	0.023	−0.015	0.028	1.097
TR9-HHH	1	1	1	0.019	0.026	0.025	−0.015	0.029	1.109

**Table 7:** Overall effect analysis with Wilks’ lambda multivariate test.

Source	Test Statistic	F-Value	Num DF	Denom DF	*p*
H	0.02406	166.151	4	122	<0.0001
W	0.09863	66.616	4	122	<0.0001
Q	0.76792	4.305	4	122	0.003
H:W	0.24373	15.640	8	122	<0.0001
H:Q	0.36910	9.851	8	122	<0.0001
W:Q	0.47660	6.840	8	122	<0.0001

**Table 8:** Univariate ANOVA outputs for D_mean_ and ρ response variables.

**Response**	**D_mean_ (mm)**			
**Source**	**Num DF**	**Sum Sq.**	**F-Value**	***p***
H	2	0.002994	221.52	<0.0001
W	2	0.000983	72.76	<0.0001
Q	2	0.000094	6.95	0.002
H:W	4	0.000485	17.94	<0.0001
H:Q	4	0.000178	6.58	<0.0001
W:Q	4	0.000247	9.16	<0.0001
**Response**	ρ (ohm*cm)			
**Source**	**Num DF**	**Sum Sq.**	**F-value**	***p***
H	2	118.784	395.86	<0.0001
W	2	54.331	181.06	<0.0001
Q	2	0.556	1.85	0.165
H:W	4	12.747	21.24	<0.0001
H:Q	4	8.203	13.67	<0.0001
W:Q	4	3.342	5.57	0.001

**Table 9:** Experimental factors, control levels, and response variable for 3D block analysis.

Control Factor	Factor Ref.	Units	Levels				Response Variables
Layer height (LH)	A	mm	0.08	0.16		0.32	Resistance (R)
Toolpath design (TP)	B	-	Linear		Zig-zag		

**Table 10:** Top view of 3D block morphology for two toolpath patterns and increasing H (scale bar = 1 mm).

	Linear	Zig-zag
3D-TR4-LMM	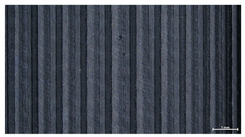	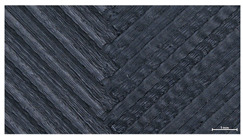
3D-TR5-MMM	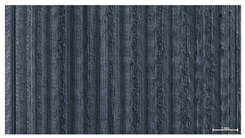	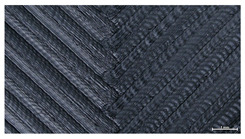
3D-TR6-HMM	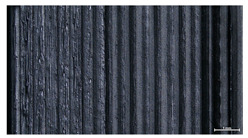	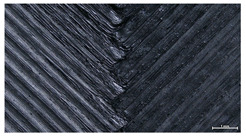

**Table 11:** Measured resistance and calculated resistivity values (total average values for 3 replicates). Reference: 40 mm filament segment prior to processing.

Test ID	Toolpath	Resistance (ohm)	SD_R_ (ohm)	Resistivity (ohm*cm)	SD_ρ_ (ohm*cm)
3D-TR4-LMM-L	Linear	333	17	19.08	0.99
3D-TR5-MMM-L		284	6	16.28	0.34
3D-TR6-HMM-L		266	10	15.23	0.58
3D-TR4-LMM-Z	Zig-zag	308	8	16.14 *	0.41
3D-TR5-MMM-Z		249	7	13.01 *	0.36
3D-TR6-HMM-Z		243	5	12.74 *	0.29
Reference	-	2063	12	12.43	0.30

Values with * are calculated with the assumption that nominal active lengths remain equal to 40 mm.

**Table 12:** Univariate ANOVA outputs for R response variable.

Response	Resistance (ohm)			
Source	Num DF	Sum Sq.	F-Value	*p*
LH	2	14,951.8	77.56	<0.0001
TP	1	3463.6	35.94	<0.0001
LH:TP	2	146.5	0.76	0.489

## Data Availability

Data are contained within the article.

## Supplementary Materials

The following supporting information can be downloaded at: https://www.mdpi.com/article/10.3390/ma16247530/s1, Table S1. List of nominal values of selected experimental factors and calculated values for G-code generation: FFF strand width (W) and height (H), volumetric flow rate (Q), FFF strand cross-section (Ac), length of filament (1.75 mm diameter) required to extrude 50 mm length of FFF strand with Ac cross-section (E), feed rate (F), and printing speed (S); Table S2. Extrudate weight values and under-extrusion percentages for the investigated nominal flow rate values; Figure S1. XZ, XY, ZY single-strand cross-sections of TR9-HHH sample in main strand volume (left) and in neck region between consecutive strands (DataViewer software v.1.2.5.7, Bruker micro-CT). Cross-section image scale: 1000 μm; Table S3. Measured resistance and cross-section values and calculated resistivity response (total average values for 3 replicates). Reference: 40 mm segment of filament prior to processing; Figure S2. Multivariate ANOVA residual plots for DD_mean_ versus H, W, Q, and 1st-order interaction terms; Figure S3. Multivariate ANOVA residual plots for ρ versus H, W, Q, and 1st-order interaction terms; Figure S4. Univariate ANOVA residual plots for DD_mean_ versus H, W, Q, and 1st-order interaction terms; Figure S5. Univariate ANOVA residual plots for ρ versus H, W, Q, and 1st-order interaction terms.
