# Supplementary material for: A Methodological Framework for Assessing the Influence of Process Parameters on Strand Stability and Functional Performance in Fused Filament Fabrication
Source: Materials (Basel). 2023 Dec 6;16(24):7530. doi: 10.3390/ma16247530 (PMC10744508; doi:10.3390/ma16247530)
Supplement: Supplementary file 1 [file materials-16-07530-s001.zip › materials-2734328-supplementary.pdf]

materials

MDPI

Supporting Information (SI)

A methodological framework for assessing the influence of process parameters on strand stability and functional performance in Fused Filament Fabrication

Eleni Gkartzou [1], Artemis Kontiza [1†], Konstantinos Zafeiris [1†], Elena Mantzavinou [1] and Costas Charitidis [1,*]

[1] Research Lab of Advanced, Composite, Nano-Materials and Nanotechnology (R-NanoLab), School of Chemical Engineering, National Technical University of Athens, 9 Heroon Polytechniou, GR-15780, Greece
† These authors contributed equally to this work
* Correspondence: charitidis@chemeng.ntua.gr;

Table S1. List of nominal values of selected experimental factors and calculated values for Gcode generation: FFF strand width (W) and height (H), volumetric flow rate (Q), FFF strand cross section (A_c), length of filament (1.75 mm diameter) required to extrude 50 mm length of FFF strand with A_c cross-section (E), feed rate (F) and printing speed (S).

Test ID	H (mm)	W (mm)	Q (mm^3/s)	A_c (mm^2)	E [1] (mm)	F [2] (mm/min)	S [3] (mm/s)	A	B	C
TR1-LLL	0.08	0.36	1.0			2188	37	-1	-1	-1
TR1-LLM	0.08	0.36	2.5	0.027427	0.570132	5470	91	-1	-1	0
TR1-LLH	0.08	0.36	4.0			8752	146	-1	-1	1
TR2-MLL	0.16	0.36	1.0			1152	19	0	-1	-1
TR2-MLM	0.16	0.36	2.5	0.052106	1.083162	2878	48	0	-1	0
TR2-MLH	0.16	0.36	4.0			4604	76	0	-1	1
TR3-HLL	0.32	0.36	1.0			644	11	1	-1	-1
TR3-HLM	0.32	0.36	2.5	0.093225	1.937918	1610	27	1	-1	0
TR3-HLH	0.32	0.36	4.0			2576	43	1	-1	1
TR4-LML	0.08	0.48	1.0			1620	27	-1	0	-1
TR4-LMM	0.08	0.48	2.5	0.037027	0.769692	4050	68	-1	0	0
TR4-LMH	0.08	0.48	4.0			6480	108	-1	0	1
TR5-MML	0.16	0.48	1.0			841	14	0	0	-1
TR5-MMM	0.16	0.48	2.5	0.071306	1.482284	2103	35	0	0	0
TR5-MMH	0.16	0.48	4.0			3364	56	0	0	1
TR6-HML	0.32	0.48	1.0			456	8	1	0	-1
TR6-HMM	0.32	0.48	2.5	0.131625	2.736161	1140	19	1	0	0
TR6-HMH	0.32	0.48	4.0			1824	30	1	0	1
TR7-LHL	0.08	0.72	1.0			1067	18	-1	1	-1
TR7-LHM	0.08	0.72	2.5	0.056227	1.168814	2668	45	-1	1	0
TR7-LHH	0.08	0.72	4.0			4268	71	-1	1	1
TR8-MHL	0.16	0.72	1.0			547	9	0	1	-1
TR8-MHM	0.16	0.72	2.5	0.109706	2.280527	1367	23	0	1	0
TR8-MHH	0.16	0.72	4.0			2188	37	0	1	1
TR9-HHL	0.32	0.72	1.0			288	5	1	1	-1
TR9-HHM	0.32	0.72	2.5	0.208425	4.332648	720	12	1	1	0
TR9-HHH	0.32	0.72	4.0			1152	19	1	1	1

Table S2. Extrudate weight values and under-extrusion percentages for the investigated nominal flow rate values.

Nominal Flow Rate (mm^3/s)	Extrudate Weight (g)	SD (g)	Under-extrusion (%)
2	0.594	0.004	0
4	0.588	0.005	-1
6	0.592	0.002	0
8	0.583	0.004	-2
10	0.573	0.004	-3
12	0.561	0.003	-6
14	0.536	0.012	-10
16	0.486	0.016	-18
18	0.337	0.160	-43
20	0.317	0.041	-47
22	0.256	0.017	-57
24	0.249	0.032	-58

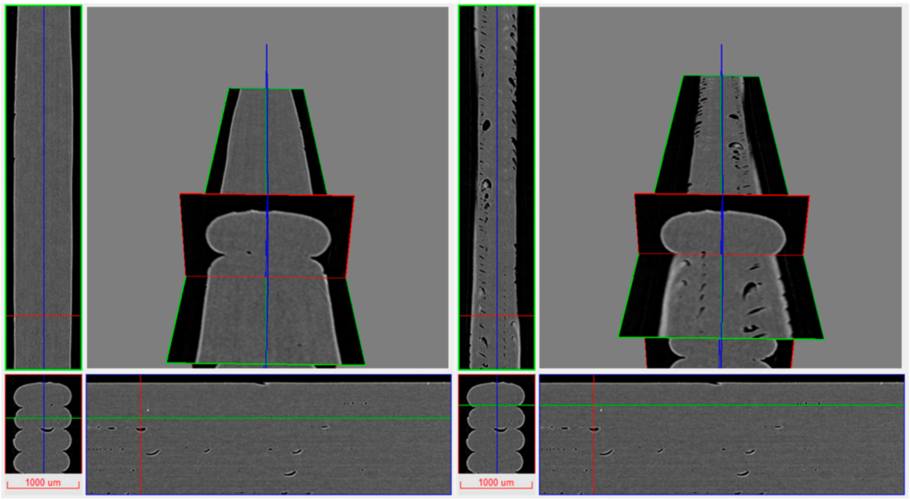

Figure S1. XZ, XY, ZY single strand cross sections of TR9-HHH sample in main strand volume (left) and in neck region between consecutive strands (DataViewer software v.1.2.5.7, Bruker microCT). Cross section image scale: 1000 um.

Table S3. Measured resistance and cross section values and calculated resistivity response (total average values for 3 replicates). Reference: 40 mm segment of filament prior to processing

Test ID	A	B	C	Cross section (mm^2)	Resistance (ohm)	SD_R (ohm)	Resistivity (ohm*cm)	SD_ρ (ohm*cm)
TR1-LLL	-1	-1	-1	0.777	4945	10	9.61	0.07
TR1-LLM	-1	-1	0	0.787	5166	15	10.16	0.28
TR1-LLH	-1	-1	1	0.824	5221	15	10.76	0.78
TR2-MLL	0	-1	-1	0.780	4434	20	8.65	0.12
TR2-MLM	0	-1	0	0.798	4383	8	8.75	0.22
TR2-MLH	0	-1	1	0.775	4427	16	8.58	0.03
TR3-HLL	1	-1	-1	0.739	4098	10	7.57	0.24
TR3-HLM	1	-1	0	0.782	3820	13	7.47	0.43
TR3-HLH	1	-1	1	0.742	4154	14	7.71	0.20
TR4-LML	-1	0	-1	1.008	3880	17	9.78	0.06
TR4-LMM	-1	0	0	1.028	3929	19	10.09	0.12

TR4-LMH	-1	0	1	1.007	4142	25	10.43	0.42
TR5-MML	0	0	-1	0.996	3671	15	9.14	0.19
TR5-MMM	0	0	0	1.013	3454	12	8.75	0.33
TR5-MMH	0	0	1	0.967	3580	12	8.65	0.36
TR6-HML	1	0	-1	0.979	3487	20	8.54	0.21
TR6-HMM	1	0	0	1.003	3194	15	8.01	0.11
TR6-HMH	1	0	1	0.971	3150	18	7.65	0.16
TR7-LHL	-1	1	-1	1.247	3982	18	12.41	0.27
TR7-LHM	-1	1	0	1.357	4134	20	14.02	0.27
TR7-LHH	-1	1	1	1.408	3589	20	12.63	0.31
TR8-MHL	0	1	-1	1.460	2793	11	10.19	0.24
TR8-MHM	0	1	0	1.457	2731	10	9.95	0.24
TR8-MHH	0	1	1	1.432	2724	12	9.75	0.34
TR9-HHL	1	1	-1	1.458	2805	22	10.23	0.29
TR9-HHM	1	1	0	1.444	2312	15	8.35	0.04
TR9-HHH	1	1	1	1.440	2269	18	8.17	0.21
Reference	-	-	-	2.405	2063	12	12.43	0.30

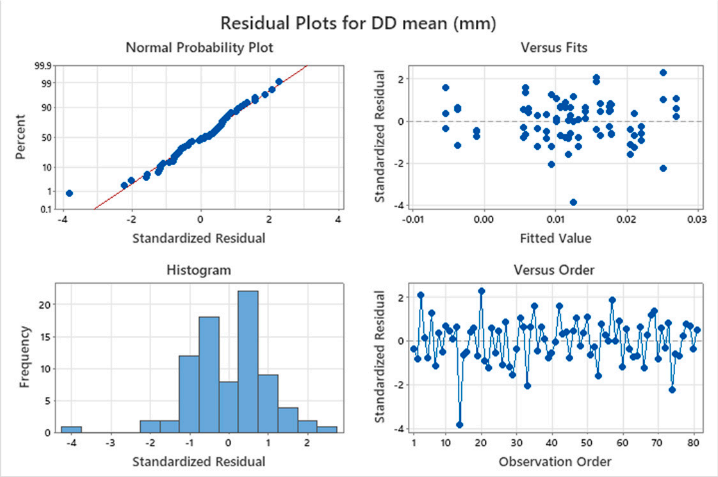

Figure S2. Multivariate ANOVA residual plots for DD_{mean} versus H, W, Q and 1st order interaction terms

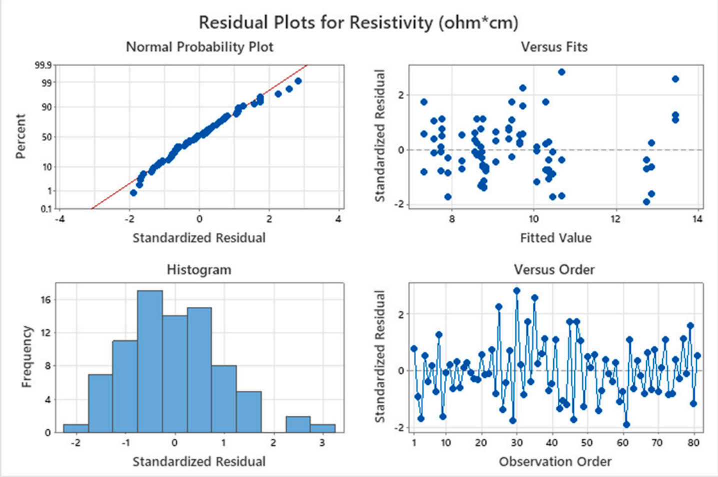

Figure S3. Multivariate ANOVA residual plots for ϱ versus H, W, Q and 1st order interaction terms

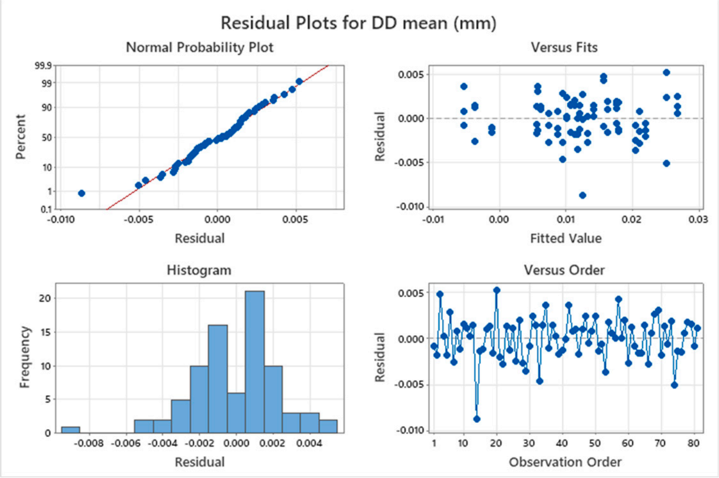

Figure S4. Univariate ANOVA residual plots for DD_{mean} versus H, W, Q and 1st order interaction terms

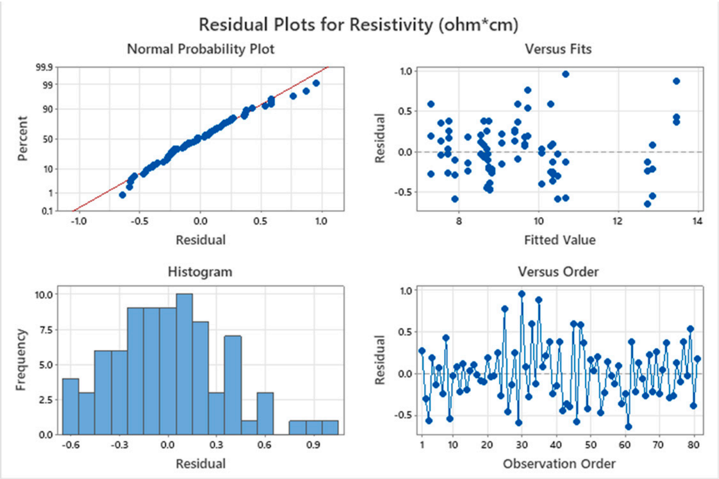

Figure S5. Univariate ANOVA residual plots for ρ versus H, W, Q and 1st order interaction terms
